# Supplementary material for: Near-infrared STED nanoscopy with an engineered bacterial phytochrome
Source: Nat Commun. 2018 Nov 12;9:4762. doi: 10.1038/s41467-018-07246-2 (PMC6232180; doi:10.1038/s41467-018-07246-2)
Supplement: Supplementary file 1 — Supplementary Information [file 41467_2018_7246_MOESM1_ESM.pdf]

**Supplemental figures and tables to**

M. Kamper et al., Near-infrared STED nanoscopy with an engineered bacterial phytochrome

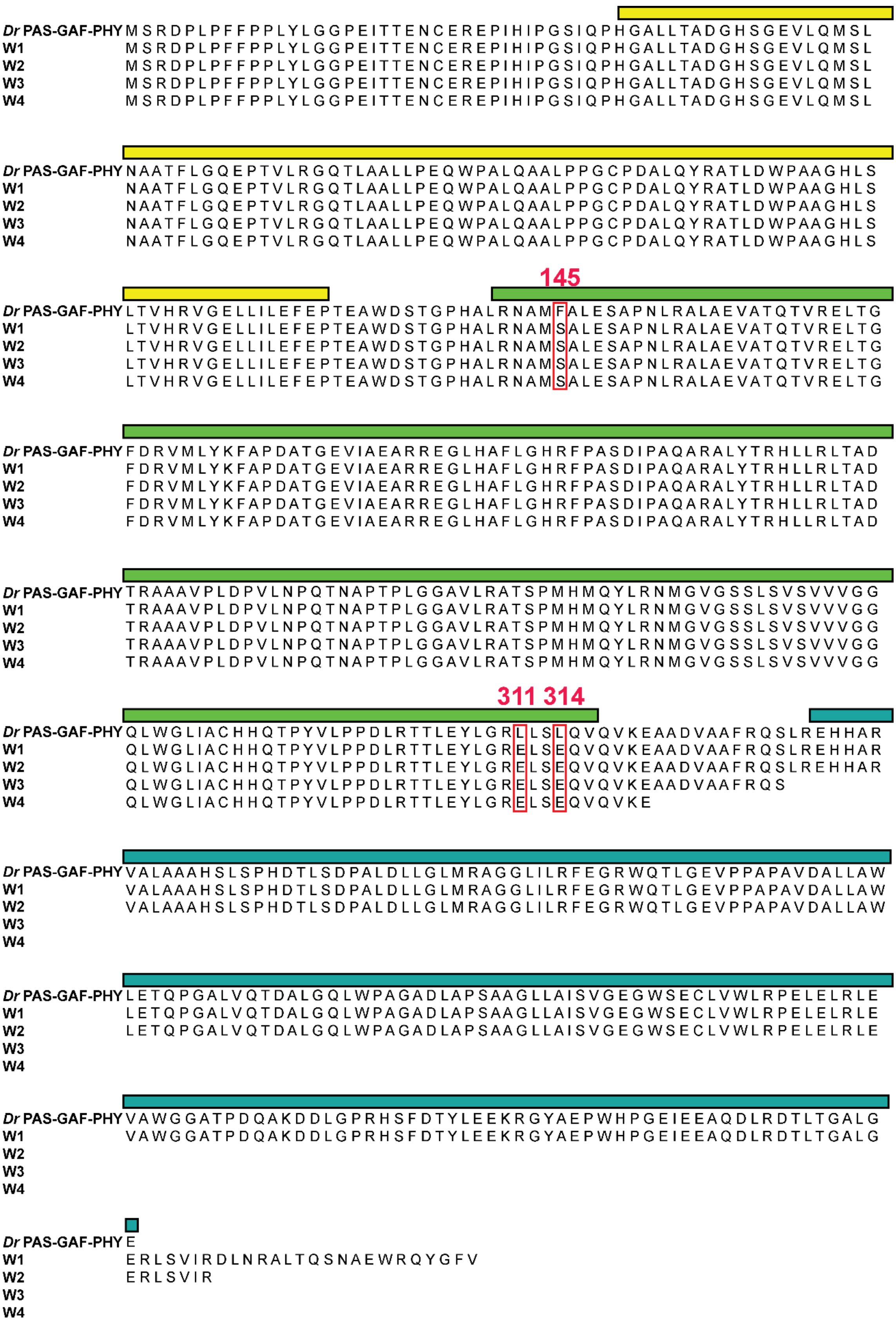


**Supplementary Figure 1** Sequence alignment against DrPAS-GAF-PHY (DR_A0050). Yellow: PAS-domain, green: GAF-domain, turquoise: PHY-domain. Red frames highlight monomerization mutations[2] (F145S, L311E, L314E).

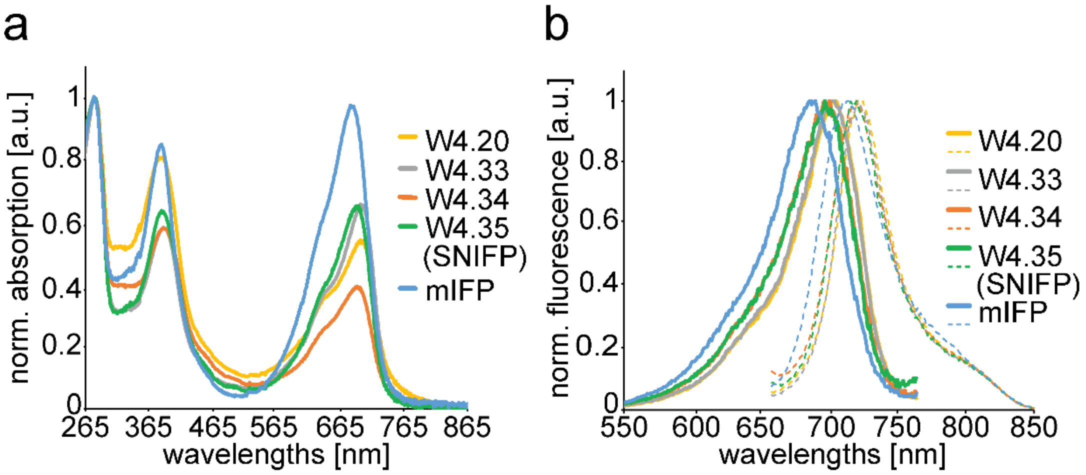


**Supplementary Figure 2** Spectra (**a**) Absorption spectra. (**b**) Fluorescence excitation (solid lines) and emission (dotted lines) spectra. All spectra were determined on purified proteins in 100 mM Tris-HCl, 150 mM NaCl, pH 7.5.

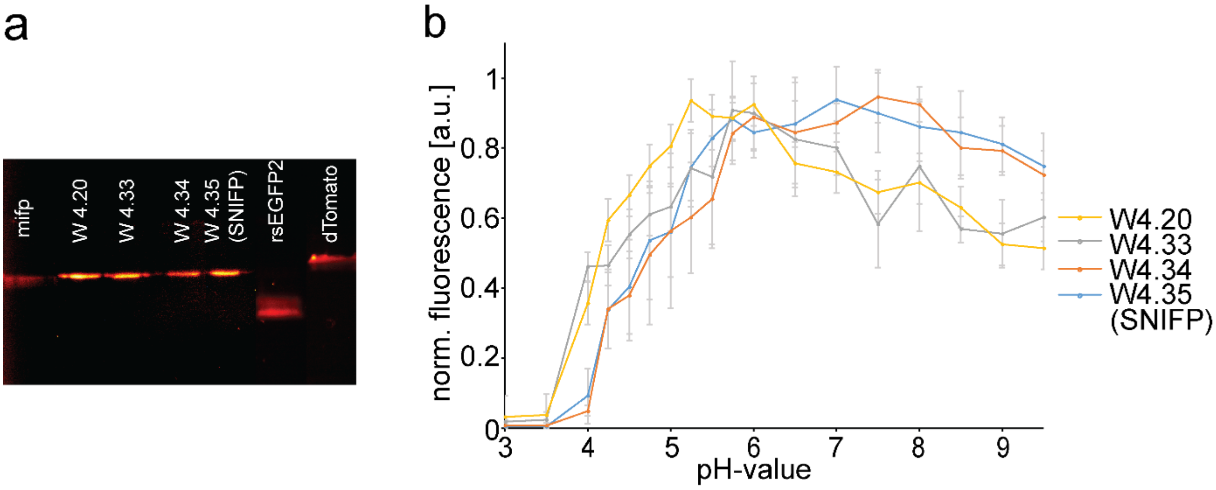


**Supplementary Figure 3** Oligomerization tendency and pH-stability. (**a**) Semi-native 15 % polyacrylamide gel (containing 0.1 % sodiumdodecyl sulfate) electrophoresis. As size standards we utilized the monomeric bacteriophytochrome mIFP[1] (~35 kDa), the monomeric fluorescent protein rsEGFP2[3] (~29 kDa) and the dimeric fluorescent protein dTomato[4] (~58 kDa). (**b**) Determination of pH-stability. The fluorescence signal was determined on purified proteins at different pH values, as indicated. Fluorescence values were normalized to the maximum. All measurements were performed in replicates (n=11 for W4.34 and W4.35; n=6 for W4.20 and W4.33). Error bars: standard deviation.

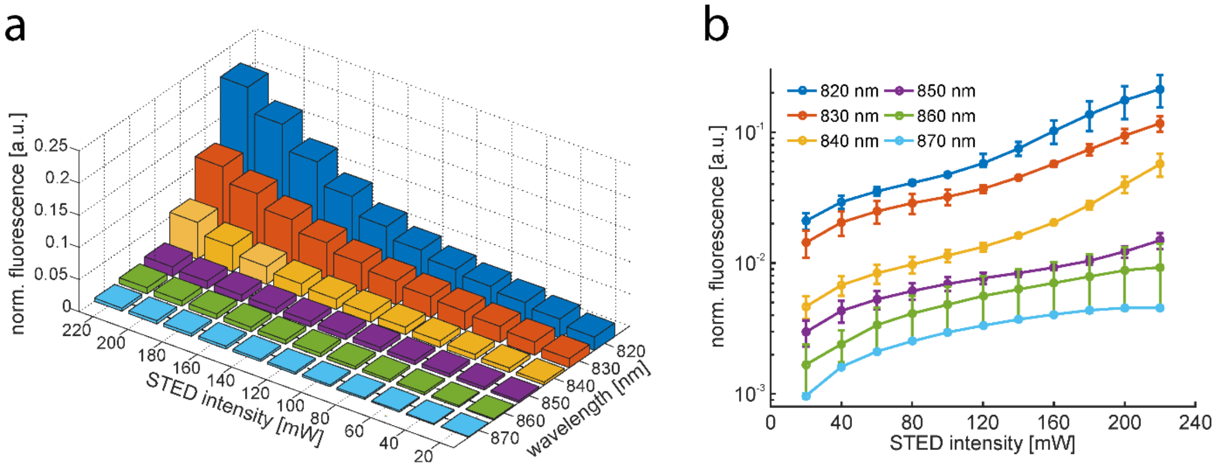


**Supplementary Figure 4** Anti-Stokes fluorescence excitation of SNIFP by the STED light. The anti-Stokes fluorescence excitation of SNIFP was measured on living Hela cells expressing VIM-SNIFP. 25 µM biliverdin was added to the medium ~two hours before imaging. We varied both the wavelength of the excitation beam (from 820 nm to 870 nm; 60 ps pulses) and the laser intensity (20 mW to 230 mW, measured at the back aperture of the objective lens). Fluorescence intensities were measured on filaments; the fluorescence signal induced by the STED beam was normalized to the fluorescence signal induced by irradiation with light of 676 nm alone (5µW at the back aperture of the objective lens). All measurements were performed in three independent replicates. (**a**) Linear and (**b**) logarithmic representation of the normalized fluorescence intensities. Error bars: standard deviation.

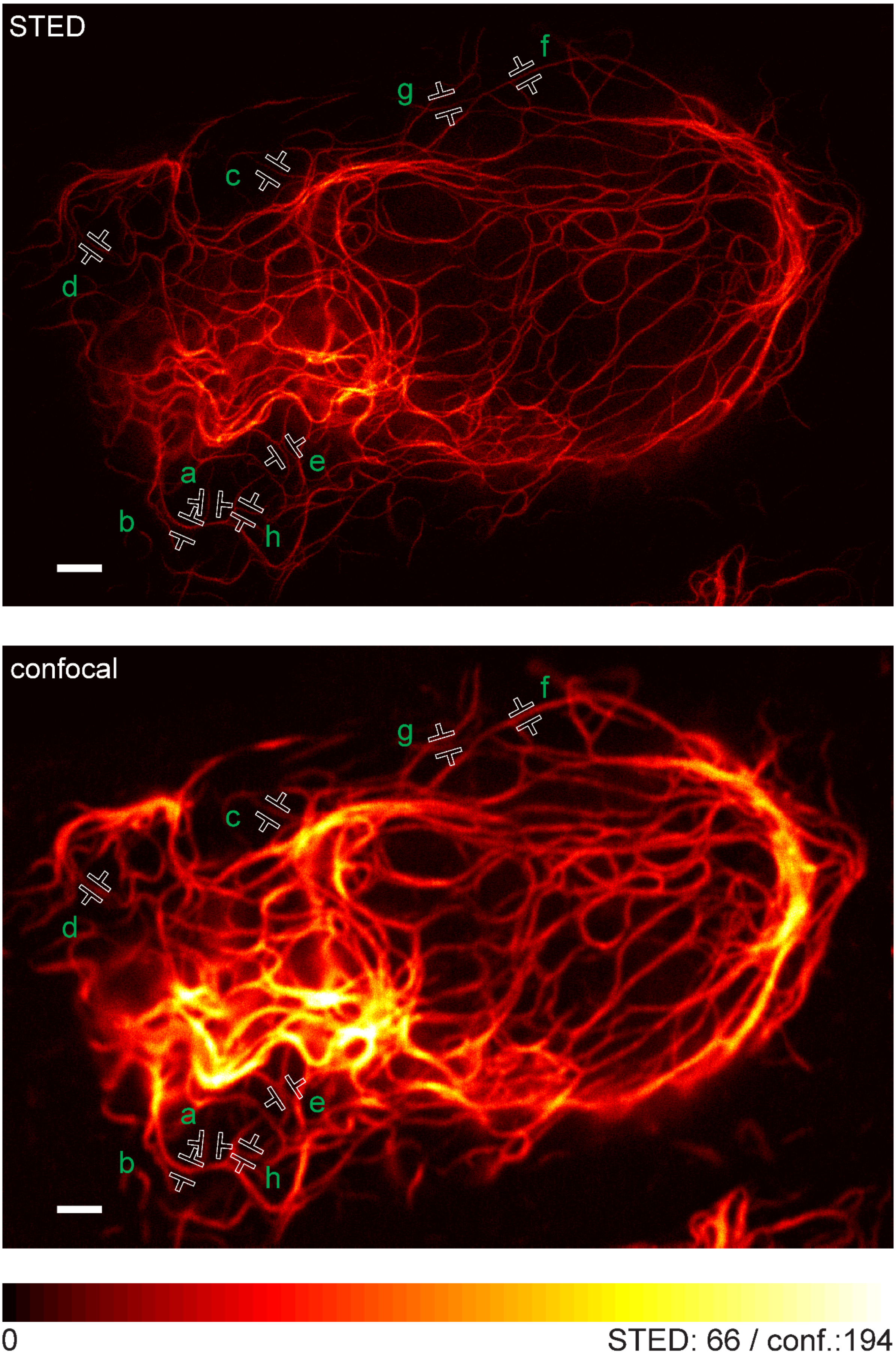
STED
confocal
a
b
c
d
e
f
g
h
0
STED: 66 / conf.:194

**Supplementary Figure 5** NIR STED nanoscopy of living HeLa cell expressing VIM-SNIFP. Shown is the same cell as in the inset of Fig. 2a. Confocal image (bottom) and corresponding STED image (top. The letters in the images (a-h) indicate the sites used to measure fluorescence intensity line profiles (see Supplementary Fig. 6). Scale bars: 2 μm.

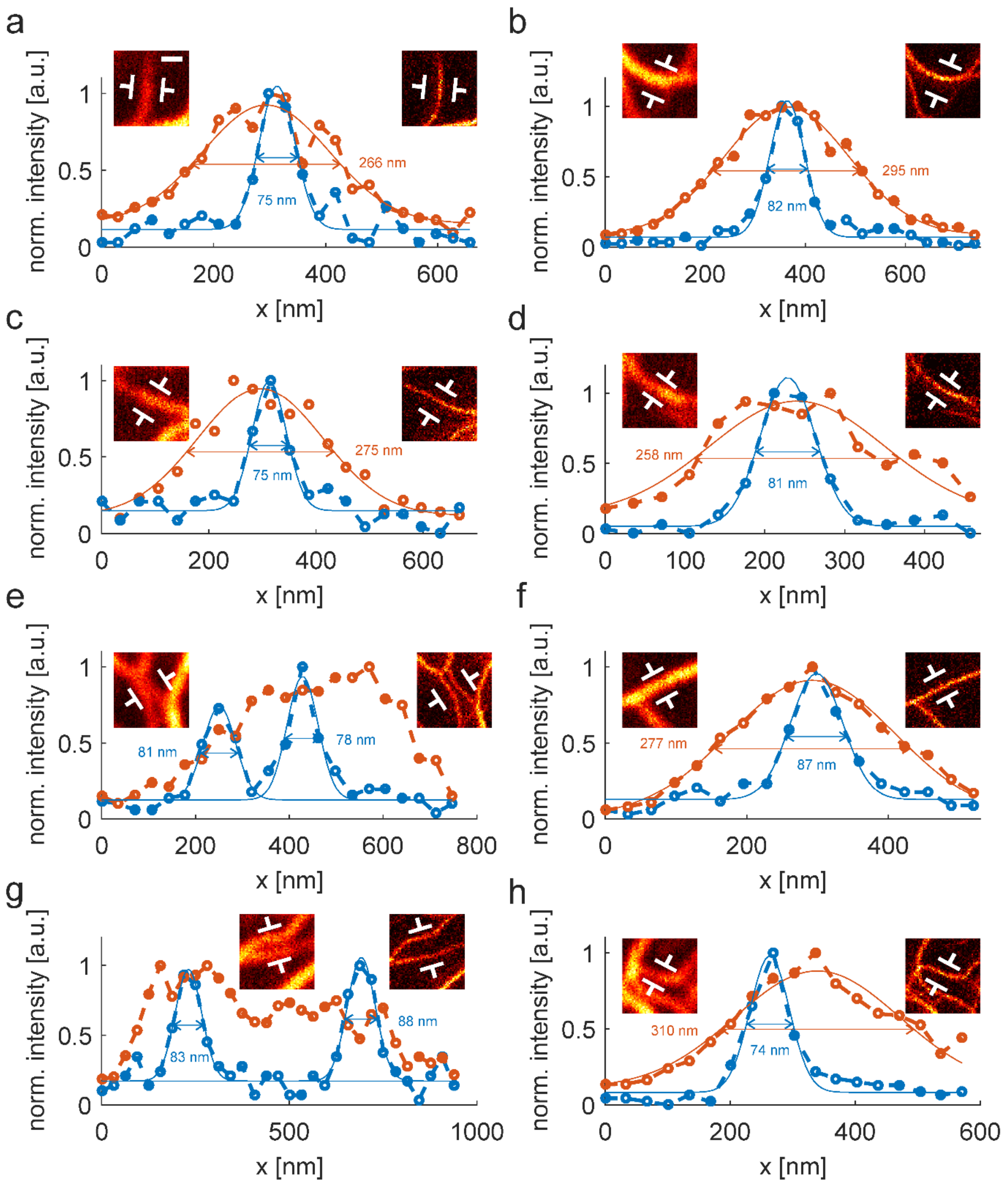


**Supplementary Figure 6** Fluorescence intensity line profiles on Vimentin filaments. Averaged line profiles were taken at the positions (a-h) indicated in Supplementary Figure 5. The line width for averaging was 90 nm (3 pixels). Solid line: The measured data were fitted with a Gaussian function. Confocal profiles are not fitted in e and g because the short distance between the two filaments made the fitting unreliable. Blue circles: STED data; orange circles: confocal data. The FWHM values were determined on the fitted function and indicated on the plots. Insets show the 1.8 µm x 1.8 µm regions where the line profiles were taken. The FWHM in the STED images is 80.5±4.9 nm (n=10) and in the confocal recordings 274±20 nm (n=8). Scale bars: 400 nm.

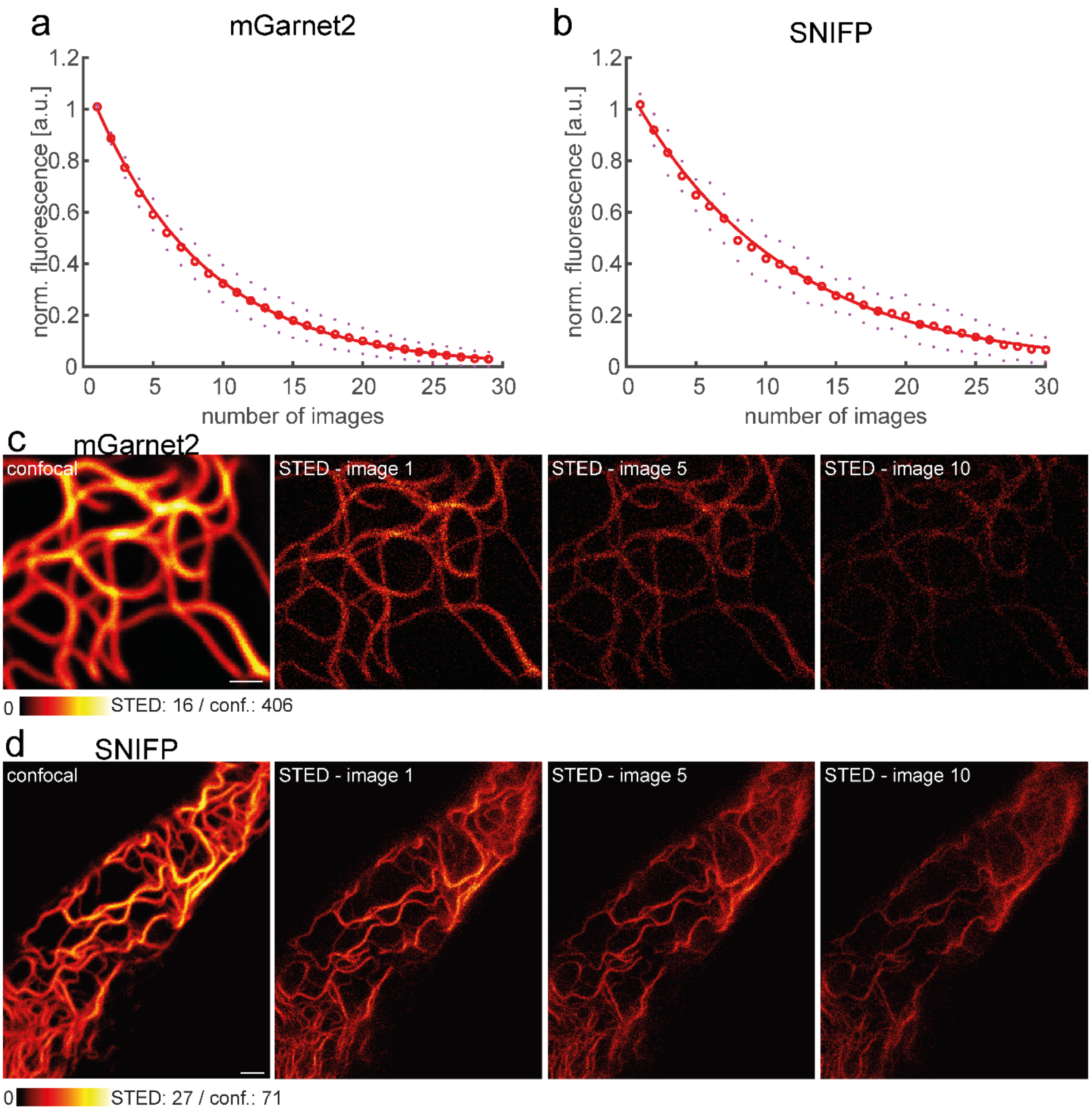


**Supplementary Figure 7** Photostability of mGarnet2 and SNIFP during STED imaging. We recorded 30 consecutive STED images at the same site in living Hela cells expressing VIM-mGarnet2 (a and c) or VIM-SNIFP (b and d). a and b, the red lines show a single exponential fit to the data (red circles) with small dots to indicate the standard deviation. The characteristic decay times (decay to 1/*e* of the initial signal) were 8 ± 2 (n=9) for mGarnet2 and 11 ± 3 (n=8) for SNIFP. Imaging conditions were choose to achieve comparable resolution. Note that SNIFP is a NIR FP, whereas mGarnet2 is a far-red FP, so that different wavelengths and imaging parameters are required for the STED recordings. c (mGarnet2) and d (SNIFP) show a confocal recording and STED images 1, 5 and 10, as indicated. In case of d, 25 µM biliverdin was added to the medium ~two hours before imaging. Scale bars c: 1 µm, d: 2 µm.

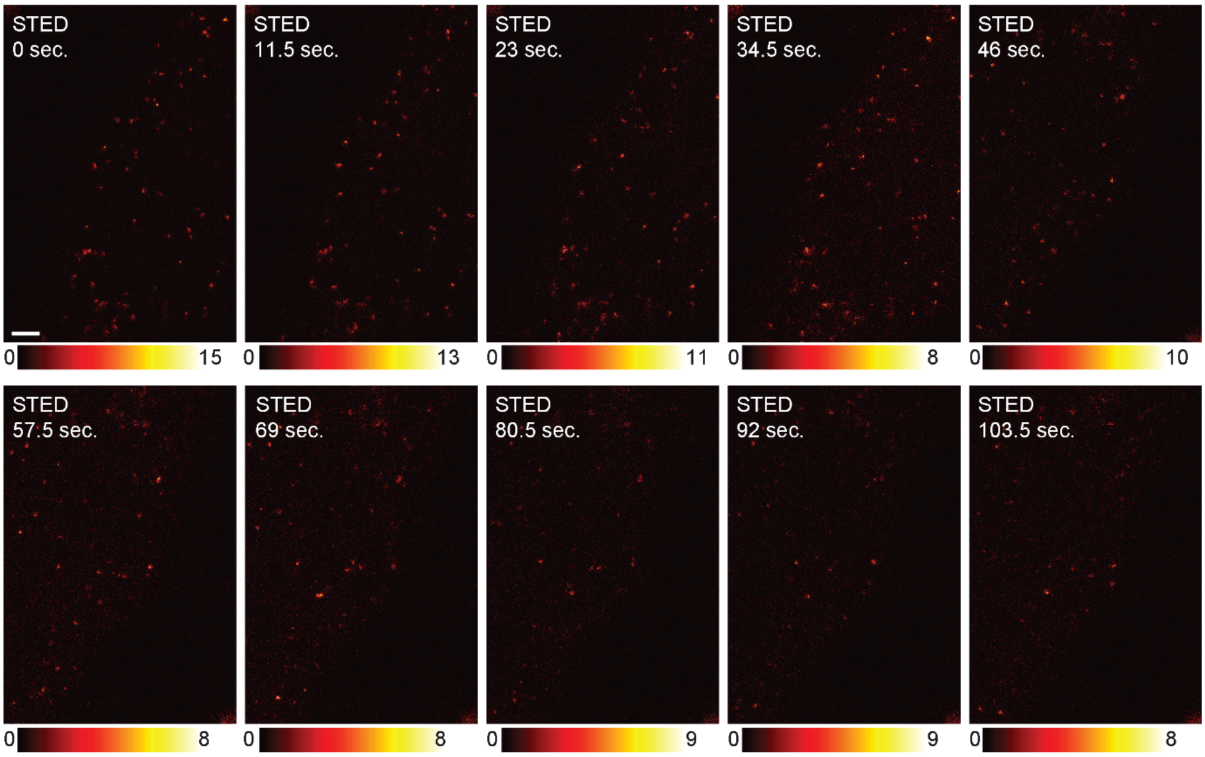


**Supplementary Figure 8** Live-cell STED nanoscopy with SNIFP targeted to the peroxisomes. Ten consecutive STED images recorded at the same site of a living HeLa cell expressing SNIFP-PTS to label the peroxisomes. Recordings were started at the indicated time points. Recording of one image took 6.5 s. 25 µM biliverdin was added to the medium ~two hours before imaging. Imaging parameters are listed in Supplementary Table 3. Scale bar: 2 µm.

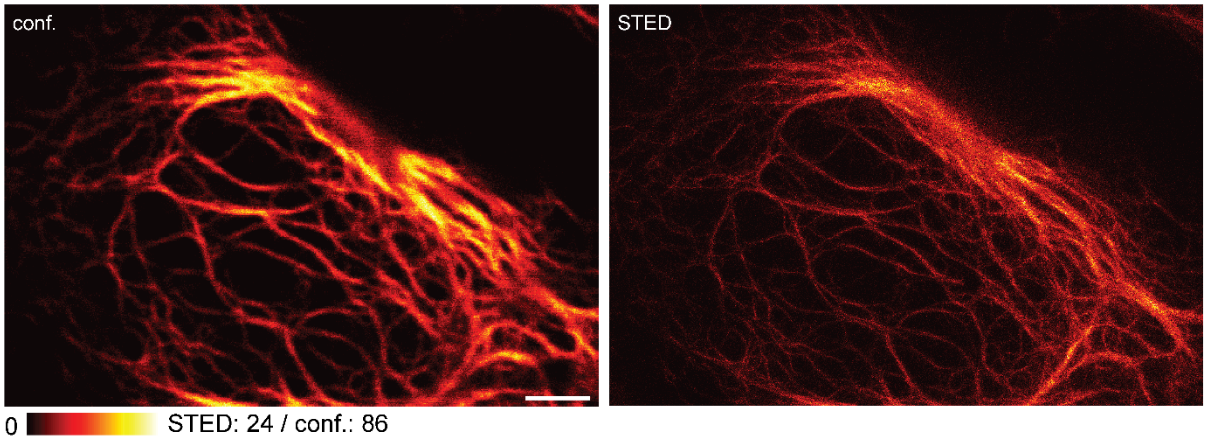


**Supplementary Figure 9** STED imaging of SNIFP without biliverdin. Confocal (left) and STED (right) imaging of a living HeLa cell expressing VIM-SNIFP without addition of biliverdin. Scale bar: 2 μm.

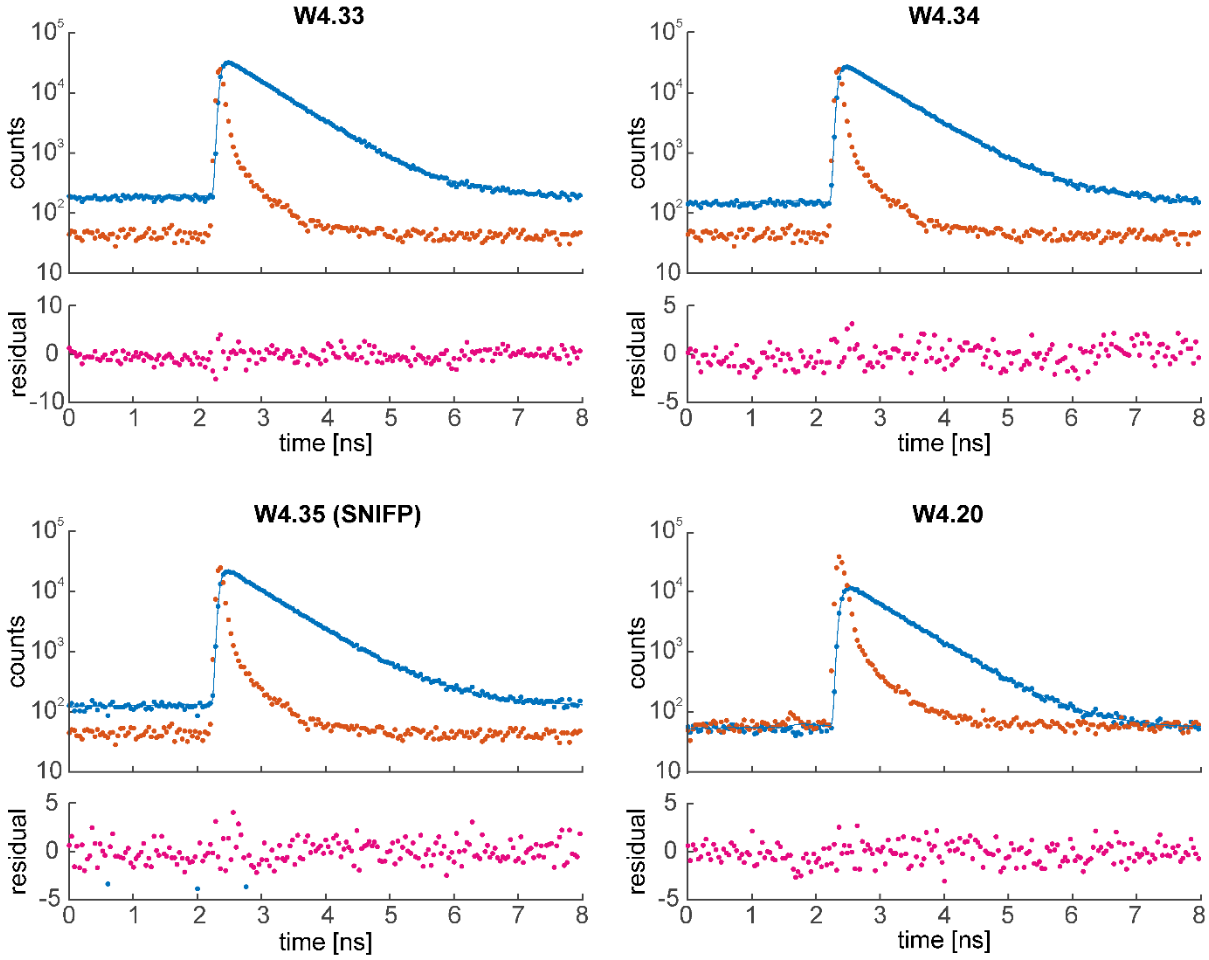


**Supplementary Figure 10** Determination of fluorescence lifetimes. The respective bacteriophytochrome variants were fused to vimentin and expressed in HeLa cells. The fluorescence lifetimes were determined in living cells at room temperature. 25 µM biliverdin was added to the medium ~two hours before imaging. The fluorescence signal was collected while scanning across Vimentin filaments. Red dots: Measured instrument response function. Blue dots: Measured decay curves. For better visualization, only every 10th data point is plotted. Solid blue line: Multi-exponential decay fit. Magenta dots: Residuals of the fit from the measured data.

| template variant | domains (aa) | cellular brightness in *E. coli* cells [% W1] |
|---|---|---|
| W1 | PAS-GAF-PHY (530 aa) | 100 |
| W2 | PAS-GAF-PHY (511 aa) | 73 ± 1 |
| W3 | PAS-GAF (331 aa) | 254 ± 4 |
| W4 | PAS-GAF (321 aa) | 359 ± 37 |

**Supplementary Table 1** Normalized cellular brightness of bacteriophytochrome variants expressed in *E. coli* cells. Protein expression was induced approximately six hours before measurement. Cells were excited with 671 nm and fluorescence was detected at 690 - 766 nm. Measurements were performed using a FACS instrument. Two independent sets of experiments were performed. Error: Standard deviation.

| W4 variant | mutations in W4 | $exc_{max}$ [nm] | $em_{max}$ [nm] | $\varphi$ [%] direct measurement | $\varepsilon$ [$M^{-1}$ $cm^{-1}$] | cellular brightness in *E. coli* cells [% W4.20] | cellular brightness in mammalian cells [% W4.20] | lifetime $\tau$ [ps] | fluorescence at pH 9 [% of max.] |
|---|---|---|---|---|---|---|---|---|---|
| mIFP* | - | 683 | 704 | - | 55000 | - | - | - | - |
| W4.20 | Y263F | 705 | 723 | 2.0 ± 0.3 | 47400 ± 1100 | 100 | 100 | 613 ± 7 | 52 ± 8 |
| W4.33 | Y263F G270R | 704 | 720 | 2.4 ± 0.5 | 59000 ± 9200 | 119 ± 10 | 133 ± 5 | 597 ± 4 | 56 ± 10 |
| W4.34 | Y263F D207L | 700 | 721 | 2.6 ± 0.6 | 83900 ± 18000 | 170 ± 13 | 228 ± 14 | 627 ± 9 | 80 ± 7 |
| W4.35 (SNIFP) | Y263F D207L G270R | 697 | 720 | 2.2 ± 0.3 | 149200 ± 19200 | 174 ± 15 | 247 ± 14 | 631 ± 11 | 81 ± 8 |

**Supplementary Table 2** Properties of NIR FPs generated in this study. All measurements were performed in replicates. Errors: Standard deviations. All data belonging to mIFP were taken from[1].

| experiment | | figure | excitation power [µW] | STED power [mW] | STED pulse length | pixel size [nm] | pixel dwell time [µs] | additional information |
|---|---|---|---|---|---|---|---|---|
| Confocal time lapse and photostability measurements | | Fig. 1 j, k | 3 or 6 | NA | NA | 60x60 | 6 | |
| STED imaging VIM-SNIFP | | Fig. 2a + Suppl. Fig. 5 | 5 | ~220 (860 nm) | ~60 ps | 30x30 | 50 | time gating |
| STED imaging SNIFP-NUP50 | | Fig. 2c | 6 | ~150 (860 nm) | ~120 ps | 30x30 | 80 | |
| 2 color STED/confocal imaging | VIM | Fig. 2d | 6 | ~190 (860 nm) | ~60 ps | 30x30 | 60 | time gating |
| | ER | | 6 | NA | NA | 30x30 | 60 | |
| STED imaging of VIM-SNIFP without BV addition | | Suppl. Fig. 9 | 5 | ~220 (860 nm) | ~60 ps | 30x30 | 20 | |
| STED time lapse and photostability mGarnet 2 (VIM-mGarnet2) | | Suppl. Fig. 7a, c | 13 (640 nm) | 53 (775 nm) | 1.3 ns | 30x30 | 30 | time gating |
| STED time lapse and photostability SNIFP (VIM-SNIFP) | | Fig. 2e Suppl. Fig. 7b, d | 5 | ~220 (860 nm) | 60 ps | 30x30 | 10 | |
| STED time lapse of SNIFP-PTS | | Fig. 2 f +Suppl. Fig. 8 | 5 | ~220 (860 nm) | 60 ps | 30 x 30 | 8 | |
| STED wavelength scan | | Suppl. Fig. 4 | 5 | 20-230 (scan) | ~60 ps | 25x25 | 4 | |
| lifetime measurement | | Suppl. Fig. 10 | 5 | NA | NA | 100x100 | 3 | |

**Supplementary Table 3** Imaging parameters.

## References


1 Yu, D. *et al.* A naturally monomeric infrared fluorescent protein for protein labeling in vivo. *Nat Meth* **12**, 763-U106, doi:10.1038/nmeth.3447 (2015).

2 Auldridge, M. E., Satyshur, K. A., Anstrom, D. M. & Forest, K. T. Structure-guided engineering enhances a phytochrome-based infrared fluorescent protein. *J Biol Chem* **287**, 7000-7009, doi:10.1074/jbc.M111.295121 (2012).

3 Grotjohann, T. *et al.* rsEGFP2 enables fast RESOLFT nanoscopy of living cells. *eLife* **1:e00248** doi:10.7554/eLife.00248 (2012).

4 Shaner, N. C. *et al.* Improved monomeric red, orange and yellow fluorescent proteins derived from Discosoma sp. red fluorescent protein. *Nat Biotechnol* **22**, 1567-1572 (2004).
