# Supplementary material for: Near-infrared STED nanoscopy with an engineered bacterial phytochrome
Source: Nat Commun. 2018 Nov 12;9:4762. doi: 10.1038/s41467-018-07246-2 (PMC6232180; doi:10.1038/s41467-018-07246-2)
Supplement: Supplementary file 3 — Reporting Summary [file 41467_2018_7246_MOESM3_ESM.pdf]

nature research

Corresponding author(s): Stefan Jakobs

Life Sciences Reporting Summary

Nature Research wishes to improve the reproducibility of the work that we publish. This form is intended for publication with all accepted life science papers and provides structure for consistency and transparency in reporting. Every life science submission will use this form; some list items might not apply to an individual manuscript, but all fields must be completed for clarity.

For further information on the points included in this form, see Reporting Life Sciences Research. For further information on Nature Research policies, including our data availability policy, see Authors & Referees and the Editorial Policy Checklist.

Please do not complete any field with "not applicable" or n/a. Refer to the help text for what text to use if an item is not relevant to your study. For final submission: please carefully check your responses for accuracy; you will not be able to make changes later.

Experimental design

1. Sample size

Describe how sample size was determined.

No statistical method was used to determine sample size. Sample sizes were chosen based on previous experience and standards in the field. In case of the FACS measurements, the sample size was dictated by the experimental procedure.

2. Data exclusions

Describe any data exclusions.

No data were excluded from the analysis.

3. Replication

Describe the measures taken to verify the reproducibility of the experimental findings.

All experiments were performed in independent replicates. The number of replicates are provided in the material and methods section or in the figure captions. Mean values and errors (standard deviation) are provided, when appropriate.

4. Randomization

Describe how samples/organisms/participants were allocated into experimental groups.

Randomization of the data was not required, as precise physical values such as absorption or emission spectra were determined. These values are independent from the observer.

5. Blinding

Describe whether the investigators were blinded to group allocation during data collection and/or analysis.

Blinding of the investigators was not required, as precise physical values such as absorption or emission spectra were determined. These values are independent from the observer.

Note: all in vivo studies must report how sample size was determined and whether blinding and randomization were used.

6. Statistical parameters

For all figures and tables that use statistical methods, confirm that the following items are present in relevant figure legends (or in the Methods section if additional space is needed).

n/a	Confirmed	
☐	☒	The exact sample size (*n*) for each experimental group/condition, given as a discrete number and unit of measurement (animals, litters, cultures, etc.)
☐	☒	A description of how samples were collected, noting whether measurements were taken from distinct samples or whether the same sample was measured repeatedly
☐	☒	A statement indicating how many times each experiment was replicated
☒	☐	The statistical test(s) used and whether they are one- or two-sided<br>*Only common tests should be described solely by name; describe more complex techniques in the Methods section.*
☒	☐	A description of any assumptions or corrections, such as an adjustment for multiple comparisons
☒	☐	Test values indicating whether an effect is present<br>*Provide confidence intervals or give results of significance tests (e.g. P values) as exact values whenever appropriate and with effect sizes noted.*
☐	☒	A clear description of statistics including central tendency (e.g. median, mean) and variation (e.g. standard deviation, interquartile range)
☐	☒	Clearly defined error bars in all relevant figure captions (with explicit mention of central tendency and variation)

See the web collection on statistics for biologists for further resources and guidance.

Software

Policy information about availability of computer code

7. Software

Describe the software used to analyze the data in this study.	FACS data were analyzed using FlowJo 7.5.5. and exported in Microsoft Excel 2016.<br>Spectra and QY-data were analyzed using Microsoft Excel 2016.<br>Data recorded with the STED microscope were analyzed using Matlab.

For manuscripts utilizing custom algorithms or software that are central to the paper but not yet described in the published literature, software must be made available to editors and reviewers upon request. We strongly encourage code deposition in a community repository (e.g. GitHub). *Nature Methods* guidance for providing algorithms and software for publication provides further information on this topic.

Materials and reagents

Policy information about availability of materials

8. Materials availability

Indicate whether there are restrictions on availability of unique materials or if these materials are only available for distribution by a third party.	No unique materials were used.

9. Antibodies

Describe the antibodies used and how they were validated for use in the system under study (i.e. assay and species).	No antibodies were used.

10. Eukaryotic cell lines

a. State the source of each eukaryotic cell line used.	U2OS (ATCC HTB-96) cells were obtained from the European Collection of Authenticated Cell Cultures (ECACC 92022711). This cell line was derived in 1964 from a moderately differentiated sarcoma of the tibia of a 15 year old girl.<br>Hek 293 (Human Embryo Kidney) cells were previously described in Ta, H., J. Keller, M. Haltmeier, S. K. Saka, J. Schmied, F. Opazo, P. Tinnefeld, A. Munk, S. W. Hell : "Mapping molecules in scanning far-field fluorescence nanoscopy". Nature Commun. 6, 7977.<br>HeLa (ATCC CCl-2) cells were obtained from the American Type Culture Collection. This cell line was derived from cervical cancer cells taken from Henrietta Lacks.
b. Describe the method of cell line authentication used.	The cell lines were not authenticated.
c. Report whether the cell lines were tested for mycoplasma contamination.	All cell lines were tested for mycoplasma contamination and found negative.
d. If any of the cell lines used are listed in the database of commonly misidentified cell lines maintained by ICLAC, provide a scientific rationale for their use.	No cell lines used in this study were found in the database of commonly misidentified cell lines that is maintained by ICLAC and NCBI Biosample.

Animals and human research participants

Policy information about studies involving animals; when reporting animal research, follow the ARRIVE guidelines

11. Description of research animals

Provide all relevant details on animals and/or animal-derived materials used in the study.	No animals were used in this study.

Policy information about studies involving human research participants

12. Description of human research participants

Describe the covariate-relevant population characteristics of the human research participants.	This study did not involve human research participants.
